# Implementation of the polluter pay’s principle in tobacco control in the UK: a stakeholder analysis

**DOI:** 10.1186/s12889-023-17219-w

**Published:** 2023-11-17

**Authors:** Marissa J. Smith, Chris Patterson, Christina Buckton, Shona Hilton

**Affiliations:** MRC/CSO Social and Public Health Sciences Unit, University of Glasgow, Clarice Pears Building, 90 Byres Road, Glasgow, G12 8TB UK

**Keywords:** Tobacco control policies, Polluter pay, Public health

## Abstract

**Background:**

The polluter’s pay principle (PPP) aims to internalise external costs and assign liability to the polluter for the harmful cost of their products to society. Tobacco companies continue to manufacture and sell harmful cigarettes, earning billions in profits each year from these products. Meanwhile, governments and their people are left to ‘clean up’ and deal with the detrimental health consequences. This paper explores with expert stakeholders how the PPP could be implemented within the context of tobacco control in the United Kingdom (UK).

**Methods:**

Twenty-four semi-structured interviews and two follow-up discussion groups were conducted with UK and international experts on tobacco control, public health, economics, or law from the academic, public, private and third sector. Participants considered the facilitators and barriers to implementing the PPP to tobacco control in the UK. Thematic analysis was employed, aided by NVivo 12, and data were compared to examine the views expressed by the different types of experts.

**Results:**

Stakeholders favoured the implementation of the PPP in the context of tobacco control and indicated that it could be acceptable and feasible to implement and that it would likely have support from policymakers and the public alike. Stakeholders unanimously agreed that any legislation and administration should be free from tobacco industry influence; however, differences arose concerning who should oversee the implementation.

**Conclusion:**

The PPP from environmental law was predominantly seen as an approach that could be usefully applied to the tobacco industry. However, there is no one size fits all template, therefore its implementation would need to be adapted to fit the UK context.

**Supplementary Information:**

The online version contains supplementary material available at 10.1186/s12889-023-17219-w.

## Background

Globally tobacco kills more than 8 million people per annum [1] including nearly 100,000 deaths in the United Kingdom (UK) [2, 3]. The devastating effects of tobacco on the health of users are well known, as are the associated economic costs, which equate to approximately US$1·4 trillion per year. [4, 5]. In the UK, the economic costs raised from tobacco sales and from excise duty on tobacco sales continues to be substantially lower than the health costs of smoking [3, 6]. While UK smoking prevalence has declined in response to tobacco control action [7–9], the smoking inequality gap has grown [8]. In 1974, 45% of the UK adult population were cigarette smokers compared to 12.9% in 2022 [9, 10]. Action on Smoking and Health (ASH) state that in England around 1 in 3 people living in social housing smoke and 1 in four 4 people in routine and manual occupations smoke compared to significantly lower rates among home owners and those in managerial and professional occupations [11] Payne et al. [12] suggest that if nobody smoked there could be 61% fewer cases of cancer linked to deprivation in England, which is equivalent of around 27,200 deprivation-linked cases per year, down to around 16,500 each year. However, the environmental impact of the tobacco industry and tobacco use are less discussed [4]. The World Health Organisation (WHO) ‘*Tobacco: Poisoning our planet’* report [13] details the extent to which the tobacco industry affects the environment, including depletion of water sources, land clearance and deforestation, and CO_2_ emissions [13]. While the tobacco industry may be seen to have taken some steps, including the use of biodegradable cigarette filters and reusable options for electronic nicotine delivery systems (ENDS) [4, 5], these approaches should be regarded with caution. Biodegradable filters have not been widely introduced, would not address all the environmental impacts of cigarette butts [14, 15], and may be used by the industry to “greenwash” consumers and non-consumers to believing that filtered cigarettes are less harmful without plastic in their filters [16]. Research has stated that the tobacco industry should pay taxes to provide funds to specialists rather than being involved in carrying out waste schemes, as the schemes can used as a way for the industry to improve their image, gain recognition from sustainability groups and make connections with policymakers [17, 18], which is against the WHO Framework Convention Tobacco Control (FCTC) [19]. In relation to ENDS, disposable electronic cigarettes (e-cigarettes) pose a substantial threat to the environment as they have been intentionally designed to be single-use and are (largely) non-biodegradable and poorly recyclable [20, 21]. Studies have raised concerns about the manufacturing and distribution process for e-cigarettes, highlighting that they may be even more damaging than for cigarettes, given the number of constituent parts [22]. In addition, it is very difficult to recycle e-cigarettes [23, 24], therefore, is against the ‘reduce’ part of the ‘reduce, reuse and recycle’ WHO guidance on health and environment [25]. Therefore, more robust approaches are required to end the environment and health harms associated with tobacco use.

One policy option that has been considered in relation to tobacco control is imposing the polluter’s pay principle (PPP). The PPP, a commonly accepted practice in environmental law, aims to assign liability to the polluter for the harmful cost of their products to society (i.e., internalise external costs). In the case of tobacco control, it would mean that the tobacco industry should bear the costs of measures to reduce the harms (both health and environmental) [26, 27] caused by tobacco. Under this approach, a levy on tobacco companies could be used to fund WHO FCTC [19]implementation [26, 27]. Several countries including France and Spain, and cities such as San Francisco (California, USA), have followed the environmental aspect of PPP in relation to tobacco control [4]. In Europe, the Single-Use Plastic Directive [28] covers tobacco with plastic filters, requiring Member States to establish Extended Producer Responsibility (EPR) schemes. This ensures that producers of tobacco products with plastic filters cover the costs of awareness raising measures, pollution clean-up, data gathering and reporting, and the costs of waste collection for those products discarded in public collection systems [28]. This form of the PPP approach is being considered in England and it would require the tobacco industry to pay the full disposal costs of tobacco waste products, ensuring the industry takes sufficient financial responsibility for the pollution its products create [29]. The rise in disposable e-cigarettes and single-use pod-style devices, such as Juul, are enhancing the tobacco waste problem. The task of separating and retrieving the components in e-cigarettes is best fulfilled by the companies that produce them, the tobacco industry. Implementation of EPR schemes and end-of-life buyback initiatives for electronic products would limit inappropriate disposal of hazardous materials [30]. With end-of-life- buy back initiative the tobacco industry would be responsible for establishing and publicising end-of-life buyback programmes to collect their used products, avoiding littered or inappropriately discarded e-waste and other hazardous materials. In 2019, the UK Government published a green paper titled *“Advancing our Health: prevention in the 2020s”*, which discussed interventions to improve population health [31]. One intervention discussed in relation to tobacco control is the PPP. The UK Government’s report [31] suggests that the funds raised from this approach would be used to fund stop-smoking services, in particular for those groups in most need (e.g., pregnant women, social renters, people living in mental health institutions, and those in deprived communities); and to supress the illicit tobacco market by improving trading standards enforcement [31]. Based on Callard’s [27] estimates of transnational tobacco companies’ profits, a 1% tax on profits could yield $200 million per year towards FCTC implementation [26].

The UK and devolved nations governments have proposed targets to reduce adult smoking prevalence to 5% by 2030 and 2034, respectively [32, 33]. However, in order to meet these proposed targets prevalence rates need to decline at a much faster rate [34] which may require additional tobacco control measures. This paper explores with expert stakeholders how the PPP in relation to health harms could be implemented within the context of tobacco control in the UK.

## Methods

This section closely follows the methodological approach detailed in [3]. We conducted semi-structured interviews and follow-up discussion groups with expert stakeholders to explore how the PPP in relation to health harms could be implemented within the context of tobacco control in the UK.

### Interviews

We used a purposive sampling approach to target UK and international experts in tobacco control regulation, public health, economics, or law from the academic, public, private and third sector. The research team collated a list of potential participants from each of the categories of experts stated above. Policy officers at Cancer Research UK (CRUK) also supplied a list of relevant experts, which was cross-referenced against the research team’s list to produce a final list of potential participants. Alongside purposive sampling, snowball sampling was also used to identify additional participants who were not included in the original collated list. Each individual on the list (n = 40) was approached by emails and provided with an information sheet. Interested participants were contacted by a member of the research team to arrange a suitable time and mode (Microsoft Teams or Telephone) for the interview.

Of the twenty-four experts who agreed to participate, 18 were based in the UK, four in the United States of America (USA), and two in South Africa. Table 1 illustrates the distribution of participants by the sector in which they primarily worked and their principal topic of work. The economists and legal scholars were all people who were (or had) actively been involved in tobacco control through those disciplines. Participants classified as ‘public health’ were those who work and publish within the broader area of public health. The ‘other’ column refers to participants from public health organisations who had involvement in tobacco control policy and people from academia who did research and advocacy around tobacco control, but not from a legal or economic position.

Based on a review of international academic and grey literature on tobacco control funds and PPP a semi-structured interview schedule (Appendix A) was developed which covered key areas, including the potential value of PPP, disbursement of funds, raising of funds, advocacy, communication and legislation and contextual factors (e.g., who should oversee implementation). The interviews were conducted between September 2020 and January 2021 by CP and CB. One interview was conducted by telephone and the remaining 23 interviews were conducted using Microsoft Teams video meetings. The interviews lasted between approximately 45 and 60 min, all were recorded and transcribed verbatim.

**Table 1:** Sample composition by primary sector of work and primary area of expertise

Primary sector	Professional disciplinary approach to tobacco control			Total
	Economics / Law	Public Health	Other	
Academia	6	3	0	**9**
Public sector	0	4	3	**7**
Third sector*	1	1	5	**7**
Private sector	1	0	0	**1**
**Total**	**8**	**8**	**8**	**24**

*Comprises of non-governmental and non-profit-making organisations (e.g., charities and voluntary groups)

## Discussion groups

In March 2021, two follow-up online discussion groups were conducted by CB and CP with nine individuals using Microsoft Teams. Participants were selected for these follow-ups based on their sectorial expertise and to represent key disciplines. The first discussion group included three third sector professionals with expertise spanning tobacco control and public health advocacy and two academic economists. The second discussion group included two public sector professionals with roles in tobacco control and public health policy and two academics with expertise in law and public health. The aim of these groups was to consider the synthesis of views from the interviews on the potential value of implementing the PPP in relation to health harms in tobacco control in the UK and to identify key considerations for policy design. Each discussion group lasted two hours, and group discussions were recorded for later checking against the minutes.

### Analysis

We conducted thematic analysis of the data from the interview transcripts and discussion group minutes. The process followed Braun and Clarke’s [35] six-phase framework for thematic analysis. All of the research team read and re-read the transcripts to become familiar with the data, and then SH iteratively constructed a coding frame to enable consistent organisation of relevant data. NVivo was used to organise categories based on inductive themes that emerged from close reading of the data to capture of both areas of agreement and less typical perspectives across a range of categories. The discussion group recordings and minutes were cross-compared with the interview coding frame to confirm and expand on codes relating to recommendations for policy design of implementing the PPP in relation to health harms in tobacco control in the UK.

Ethical approval for this study was granted by the College of Social Sciences Research Ethics Committee at the University of Glasgow (reference 400,190,213).

## Results

The results are presented in accordance with the inductive coding categories developed during the analysis stage.

### What is the potential value of implementing the PPP?

There was general agreement between participants that there is potential value to implementing the PPP in the context of tobacco control and that this could be a valuable revenue for raising predictable and reliable funds direct from the tobacco industry. In addition, it would help hold the tobacco industry to be held more accountable for the damage they cause to society, with one participant stating:

*“There’s some sort of nice symmetry about money from the tobacco industry being used to improve or solve some of the problems it creates.” (P05, third sector, public health)*

Participants typically favoured the polluter pays metaphor, and many indicated that it could be persuasive to both policymakers and the public:

*“I think it’s a slam dunk. I don’t think there can be any question about that. The polluter pays phrase was the one thing that got into the government’s Green Paper [Advancing our health: prevention in the 2020s] on this. So, while they…you know, calling it an industry levy didn’t wash, they did say they were willing to look at ways of making the polluter pay.” (P18 public sector, public health)*

*“I think that’s how you’d sell it to the public, and that they are essentially polluting, so the whole polluter pays thing, you know, so, you know, evocative, emotive language is probably so so, you know, tobacco industry is a polluter”. (P15, third sector, other)*

However, some participants highlighted the need for clarity, particularly surrounding the term ‘polluters pay’ and its definition, when applied to tobacco control:

*“We’d need to be very, very careful in the communications about this, particularly in relation to who is the polluter. Because at the very beginning of this I suppose it is possible for someone to put out misinformation that would say they’re after you because you’re putting the tobacco smoke into the air by smoking, so you’re the polluter. […] You can find some references to polluter pays and public health but not very many. It’s mainly people shouting saying we should have a polluter pays principle for public health but there’s not much to say that’s happening. So, I think we’d need to really educate people what we mean by it and communicate by it.” (P11, third sector, other)*

In addition, several participants questioned PPP’s applicability to tobacco due the potential for it to cause confusion due the industry’s shift toward harm reduction and the development of harm reduction products (such as ENDS):

*“I think it’s problematic, maybe this is just the lens that I look through it, but to me when you talk about polluter pays, you’re talking about the person who creates the negative consequences pays for the negative consequences. And I think one of the things that industry is moving towards is this harm reduction, harm minimisation narrative. And so as soon as you start defining something based on the outcomes, they will argue with you a lot and they will try and dispute any evidence base and they will try to create their own evidence base.” (P05, third sector, public health)*

While participants were predominantly supportive of the polluter pays metaphor, some questioned its applicability to tobacco due the potential for it to cause confusion, and the complexity that harm-reduction products introduce.

### What are the potential industry arguments?

Participants discussed a range of arguments that industry are likely to use in response to implementing the PPP, and some suggested counter-arguments. Participants typically expected industry to react negatively, both directly and indirectly through surrogates:

*“You would get fierce opposition to anything that would restrict their operation and particularly anything that would incur additional costs, so yeah, you get the idea, it’s not something they would embrace without a fight.” (P05, third sector, economist)*

One participant suggested that strong industry opposition would be reflective of the likely effectiveness of a policy:

*“Well the bigger the noise the better because it shows that they’re bloody scared of it.” (P08, third sector, other)*

In contrast, two participants warned that industry has the power to shape the narrative around a policy, even if their arguments are not robust:

*“The big card they always play is the fact that we raise more in taxes than we spend on dealing with the harm which we dispute. But it doesn’t matter if you dispute it or not, if they say it, it becomes the narrative.” (P16, public sector, other)*

Several participants discussed that the industry would argue that implementing the PPP in relation to health harms is unfair and unjustified and would object about being treated differently to other unhealthy commodity industries:

*“If you worked in the tobacco industry you might say well, drinking, because drinks can kill you, can’t they? And eating unhealthy food can kill you, and stuff, as well, so, are we all polluters.” (P14, public sector, public health)*

A variety of arguments were put forward by participants that the tobacco industry are likely to use in response implementing the PPP (including the effectiveness of the policy and disfavour of the tobacco industry), and some suggested counter-arguments (including the power of the tobacco industry to shape the policy narrative).

### Who should oversee implementation?

Participants discussed who they considered should oversee the implementation of PPP for tobacco control. These discussions included consideration of what type of body should be responsible, what sources of expertise should be involved, and what stakeholders should not be allowed to exert influence. There was much agreement between participants for a transparent and independent body, to oversee PPP’s implementation:

*“I don’t think I would have a strong view as long as […] it was a transparent body that both industry and [academic] researchers and the government had trust in to operate transparently and fairly and not be unduly influenced by any stakeholders, by researchers, by industry, by government, you just need to make it an independent body.” (P05, third sector, public health)*

One public sector participant set out the need for input from a wide range of stakeholders, but also cautioned that involving too many stakeholders may be counterproductive and could negatively influence implementation.

*“There are many players in tobacco control. So there’s local Government, there’s ASH, there’s Public Health England, as it is at the moment, NHSE, Department of Health and Social Care, there’s quite a few. And the reality is you probably need input from a variety of stakeholders, but there is a worry that there might be too many cooks…too many cooks spoil the broth.” (P16, public sector, other)*

Some participants identified a need for administrative independence between devolved governments and different regions:

*“At some points in that one country might be further ahead than the other one, so we might need to spend the money more differently, you know, so there’s quite a lot of different changes. So for example in England the cessation services are provided by local authorities. In Scotland they’re provided by the NHS, so there are unique, kind of, structures in place, so I think it might be quite difficult if one organisation decides where the money’s being spent in all those devolved administrations because they might not have the on the ground feel for where it’s needed.” (P11, third sector, other)*

Participants were asked for their views on which parts of the tobacco industry should be expected to pay a potential industry charge, and how fees should be apportioned between different companies. Several participants identified the challenges of identifying which types of companies should be liable to pay an industry charge; however, few concrete solutions were suggested:

*“Is the industry the retailer or is it distribution, or is it the importers or is it, you know, the overseas owners? […] I would imagine where people who produce or distribute will find legal barriers to put up to avoid information sharing that’s potentially confidential because of its, you know, economic nature, so I would suggest retail or distribution maybe are ways to tackle that.” (P16, public sector, other)*

While some participants indicated that UK-based distributers of tobacco products should be included, participants typically agreed that the main target of a charge should be the UK-based operations of large, transnational tobacco producers as their profits are particularly high relative to other industries:

*“I’ve always said that if you want to raise money from the tobacco industry then you need to go after the companies themselves which means a charge on their profits.” (P09, academic, economics)*

Participants thought it was important that whatever body was responsible for implementation of PPP for tobacco control should be independent, transparent, and encompass a range of stakeholders.

### How should funds be disbursed?

Participants discussed the best uses of funds, with some activities seen as valuable by most participants (e.g., preventing initiation), while others were more controversial (e.g., combating illicit trade) and some were uniformly seen as poor uses of funds (e.g., school programs).

Participants typically favoured spending funds on prevention (including both preventing initiation and facilitating cessation) instead of treating the harms of smoking.

*“The bit that we struggle to fund is the preventative work. You know, people pitching up with COPD or lung cancer, for example, are getting treated; that isn’t a problem in a UK context, whereas actually we are struggling with preventative funding. So it feels to me that that’s why I would argue that very, very strongly.” (P22, public sector, public health)*

Further, participants argued that treatment is beyond the remit of tobacco control, and that funds generated from the PPP should be distinct from National Health Service (NHS) budgets:

*“The danger is that politicians think this is just another way to fill the bottomless pit that is the NHS with more money, and the point is that it’s smokers…the money is coming from smokers, because the profits come from smokers. Current smokers. And, therefore, that money should go in helping those smokers quit, and that’s what tobacco control is about. It’s not about treating them once they’re sick, it’s about supporting them to quit and preventing youth uptake.” (P23, third sector, other)*

In contrast, a minority felt that, just as recouping damages may be a valid justification for implementing the PPP fund, funding the NHS may be a valid purpose:

*“[One] argument would be, well it should go for the National Health Service. And that of course turns on how you do the calculations for the damage charge or whatever you’re calling it. To the extent the calculations are based on cost to the National Health Service, the logic is very strong that the money should go to the National Health Service.” (P02, academic, law)*

Participants presented varying attitudes to funding cessation activities. Some perceived cessation as the highest priority for funding, with many identifying limitations in existing cessation service provision:

*“I think the primary uses would be to support cessation for existing users, use that to support cessation counselling, cessation products, and then to use some of it for prevention purposes.” (P04, academic, economics)*

*“I would have thought smoking cessation services would be a good place to start given that I know some funding for those have been cut back.” (P09 academic, economics)*

In contrast, several participants saw cessation as a poor long-term investment:

*“[A healthcare professional may] say you need to spend all of the money on smoking cessation services, because they are just so chronically underfunded, as is a lot of the NHS. But that does a different thing which is that it helps people stop smoking where we missed the boat, we didn’t prevent them taking it up, so that doesn’t solve the long-term problem of tobacco use and you’re still potentially getting uptake.” (P05, third sector, public health)*

There was some disagreement over the importance of funding work to limit the illicit tobacco trade. Several participants argued that extra funding could help to address existing shortcomings in tackling illicit trade:

*“I think using some of the funds to try to deal with illicit trade in tobacco products is a good idea, step up enforcement efforts and things like that, use some of the funds to deal with under-age sales, sales to under-age kids would be appropriate.” (P04, academic, economics)*

However, the majority of participants felt that illicit trade is already policed effectively in the UK:

*“I would rank [combating illicit trade] way down the bottom because I am aware of quite an extensive literature demonstrating that this is basically a small issue blown up into a large issue by the industry and that they deliberately exaggerate the scale of the problem.” (P03, academic public health)*

Participants identified school programmes as a particularly poor use of funds, arguing that targeting adult behaviours is more effective:

*“We’ve done minimal around the school programmes and yet we’ve got the lowest youth smoking prevalence because we’ve absolutely focused our policy efforts on changing the adult world. […] I’m not saying that there isn’t a role for education in schools if it’s linked in to kind of really robust personal, social, citizenship-y kind of education, but the tobacco industry would love us to just go and run education sessions in primary schools.” (P08 third sector, other)*

*“So it’s like doing a school-based education on not smoking, which really doesn’t work. So these like evidence-based policies for youth prevention. And the best actually, the best youth prevention, is reducing the consumption among adults.” (P12, academic economics)*

Concerning the disbursement of fund, activities such as preventing initiation were considered as most valuable. Whereas activities such as combating illicit trade were more controversial and some activities, including school programs, were uniformly seen as poor uses of funds.

## Discussion

Our study explores the possible implementation of the PPP in relation to health harms in the context of tobacco control in the UK, synthesising the perspectives of professionals with expertise in tobacco control, economics and public health policy. Combining 24 in-depth expert interviews and two expert discussion groups allowed a wide-ranging examination of the state of tobacco control funds internationally, the possible routes to implementing the PPP in the UK, and the challenges that must be met in doing so. The PPP from environmental law was predominantly seen as a metaphor that could be usefully applied to the tobacco industry’s health costs, and, while participants predicted industry resistance, they typically suggested that that resistance could be overcome. Participants agreed that a transparent and independent body should oversee PPP implementation; however, if the tobacco industry state that they have trust in this body, this could potentially breech the WHO FCTC [19] as it could be regarded as the industry attempting to make connections and influence this body. Industry resistance and influence is relevant in terms of both policy acceptability and ensuring compliance with WHO FCTC [19]. Experts discussed rather than having the costs passed on to smokers, instead we should aim to directly penalise the tobacco companies who make large profit from putting lives at risk and creating immense pressure on NHS resources. In addition, if implemented correctly, the PPP approach would assist the UK and devolved nations governments’ in achieving their proposed targets to reduce adult smoking prevalence to 5% by 2030 and 2034, respectively [32, 33]. Participants highlighted the need to set clear goals and participants’ perspectives on this issue were consistent with a recent Public Health England report on fiscal and pricing policies [36], which highlights that policy success depends on policy goals. The report finds that aiming to achieve both health promotion and revenue raising objectives with the same policy can create trade-offs, but suggests that achieving this is possible when demand for a product is relatively price inelastic (i.e. the price of a product does not change even if supply or demand go up or down), as is the case with tobacco [36]. From this perspective, permitting costs to be passed on to customers and ensuring that costs are paid by industry may each be valid goals. Participants also discussed how funds should be distributed, exploring the arguments for and against investing in treatment, prevention, cessation services, combating illicit trade and school programmes. Participants exhibited broad agreement on the usefulness of funding activities such prevention and cessation services, while combating illicit trade was more controversial and school programs were uniformly seen as poor uses of funds. Conversely, research from the A Stop Smoking in Schools Trial (ASSIST) school project found educating peer leaders on tobacco in schools was beneficial [37]. Research has shown that the tobacco industry use corporate social responsibility activities (such as tidy cigarette butt campaigns) to position themselves as responsible companies with a legitimate role in society, while they continue to produce and promote their products [38, 39]. Therefore, in order for the PPP to work in relation to tobacco control, a barrier is required between tobacco industry funding and the distribution and use of the funds.

The purposive, targeted approach to sampling ensured that the research sample included a usefully broad range of professional expertise, and the depth of the interviews ensured that that expertise was represented thoroughly. Understanding the experiences and perspectives of experts within a field is useful in policy research due to the extent to which policy is constructed through the discursive engagement of different coalitions of policy actors [40]. While the interviews were valuable in producing rich data based on deep professional insights into relevant aspects of tobacco control, the one-on-one nature of the interviews meant that perspectives from one discipline were not in direct dialogue with perspectives from others. The key benefit of the discussion groups was to create informed dialogue between experts with different background and perspectives so that those perspectives could be challenged, and robust, pragmatic conclusions could be drawn. Together this data offered a valuable means of arriving at grounded policy recommendations through interdisciplinary discussion, useful in policy research due to the extent to which policy is constructed through the discursive engagement of different coalitions of policy actors [40]. Another strength was using online data collection which proved to be straightforward reduced geographical barriers to participation among world-leading experts in the UK, the USA, and South Africa. However, some limitations are worth noting. The qualitative nature of the primary research data is such that the analysis offers depth of opinion within the research sample but does not offer any predictions about the frequency of specific stances within any wider population. As such, the value of qualitative policy research is in identifying useful reasoning and novel ideas, not making generalisations about how commonplace specific opinions are. The research design was impacted substantially by the COVID-19 pandemic. Initially the primary research was intended to involve face-to-face interviews, and deliberative groups that would have been larger, longer, and more intensive than the discussion groups that replaced them. Instead, interviews were all conducted through online videoconferencing software, with the exception of one telephone call, and the scope of the deliberative groups was reduced to better allow for the videoconferencing medium. The researchers felt that the online discussion groups were effective, and any negative impacts of not being able to assemble participants in the same room were likely outweighed by the flexibility provided by not being limited by geography in determining composition of groups, enabling discussion between experts in the UK, the USA and South Africa. Beyond practical concerns, the pandemic affected the data as the primacy of the COVID-19 on the news and public health agendas meant that conversations often returned to that topic, which was frequently associated with uncertainty about the economic and political context. However, this may represent a benefit as participants were able to reflect on the extent to which the pandemic represents either an obstacle or an opportunity for policymaking in this area. Of note, participants did not discuss the environmental costs of implementing PPP in relation to tobacco control; however this was likely due to the aim of the project which was to explore the how the PPP in relation to health harms could be implemented within the context of tobacco control in the UK. As such there remains a need to explore the environmental costs of implementing PPP within the context of tobacco control. The complexity of policies and policymaking environments is such that transferring learning from one policy to a different policy is challenging [41]. As such, few participants possessed the breadth of context and knowledge to be able to present comprehensive recommendations for policy. However, this study offers new insights into an under-researched area.

## Conclusion

Tobacco is the only product that is lethal when used as intended, killing at least half of its users long-term. Tobacco companies are also highly profitable, with the pandemic appearing to have had little impact on these profits. However, there is no ‘one size fits all’ template for such fund, the structure, and operations of the fund would need to adapt to other countries to fit the culture, government ideology, and social context. Importantly, the implementation of PPP in the context of tobacco control would help meet English and Scottish targets of reducing adult smoking prevalence to 5% by 2030 and 2034, respectively.

### Electronic supplementary material

Below is the link to the electronic supplementary material.

Supplementary Material 1

## Data Availability

All data relevant to the study are included in the article or uploaded as supplementary information.

## References

[1] World Health Organisation. Tobacco: World Health Organisation. 2022 [updated 22 May 2022. Available from: https://www.who.int/news-room/fact-sheets/detail/tobacco.

[2] Action on Smoking and Health. Smoking Statistics: Action on Smoking and Health. 2021 [updated May 2021. Available from: https://ash.org.uk/resources/view/smoking-statistics#:~:text=Illness%20and%20disease,deaths%20a%20year%20in%20England.

[3] Hilton S, Smith MJ, Buckton CH, Patterson C (2022). Experts’ views on how to design a Tobacco control fund in the UK. BMJ Open.

[4] The Lancet Respiratory Medicine (2022). The hidden costs of big Tobacco. The Lancet Respiratory Medicine.

[5] Lam J, Schneider J, Shadbegian R, Pega F, St Claire S, Novotny TE (2022). Modelling the global economic costs of Tobacco product waste. Bull World Health Organisation.

[6] Branston JR, Gilmore AB (2019). The failure of the UK to tax adequately Tobacco company profits. J Public Health.

[7] Cancer Research UK. Tobacco statistics 2020 [Available from: https://www.cancerresearchuk.org/health-professional/cancer-statistics/risk/tobacco.

[8] Office for National Statistics. Adult smoking habits in the UK: 2019. 2020.

[9] Office for National Statistics. Adult smoking habits in the UK: 2022 2023 [updated 05 September 2023. Available from: https://www.ons.gov.uk/peoplepopulationandcommunity/healthandsocialcare/healthandlifeexpectancies/bulletins/adultsmokinghabitsingreatbritain/2022#:~:text=Based%20on%20APS%20data%2C%20the,20.2%25%20of%20the%20population.

[10] Office for National Statistics. Smoking (General Lifestyle Survey Overview - a report on the 2011 General Lifestyle Survey) 2013 [updated 07 March 2013; cited 2023 26 September]. Available from: https://www.ons.gov.uk/peoplepopulationandcommunity/personalandhouseholdfinances/incomeandwealth/compendium/generallifestylesurvey/2013-03-07/chapter1smokinggenerallifestylesurveyoverviewareportonthe2011generallifestylesurvey#:~:text=Consistently%2C%20men%20have%20smoked%20more,1982%20to%2013%20in%202011.

[11] Action on Smoking and Health. Smoking and health inequalties: Action on Smoking and Health. 2022. Available from: https://ash.org.uk/uploads/Smoking-and-health-inequalities-July-2022.pdf?v=1662387660.

[12] Payne NWS, Brown KF, Delon C, Kotrotsios Y, Soerjomataram I, Shelton J (2022). Socio-economic deprivation and cancer incidence in England: quantifying the role of Smoking. PLoS ONE.

[13] World Health Organisation (2022). Tobacco: Poisoning our planet.

[14] Stigler-Granados P, Fulton L, Nunez Patlan E, Terzyk M, Novotny TE. Global Health perspectives on cigarette butts and the Environment. Int J Environ Res Public Health. 2019;16(10). 10.3390/ijerph16101858 PMC6572616 31130709

[15] Slaughter E, Gersberg R, Watanabe K, Rudolph J, Stransky C, Novotny T (2011). Toxicity of cigarette butts, and their chemical components, to marine and freshwater fish. Tob Control.

[16] Evans-Reeves K, Lauber K, Hiscock R (2022). The ‘filter Fraud’ persists: the Tobacco industry is still using filters to suggest lower health risks while destroying the environment. Tob Control.

[17] Smoke Free Partnership (2022). The EU Polluter pays Principle and the Tobacco Control Legal Framework.

[18] Comité National Contre le Tabagism, editor French case study Tobacco industry tactics around Single-Use Plastics (SUP) policies and civil society efforts to countering these. WHO Conference of the Parties. 2022; Rotterdam: Comité National Contre le Tabagism.

[19] World Health Organisation (2003). WHO Framework Convention on Tobacco Control.

[20] Pourchez J, Mercier C, Forest V (2022). From Smoking to vaping: a new environmental threat?. The Lancet Respiratory Medicine.

[21] Delnevo C, Giovenco DP, Hrywna M (2020). Rapid proliferation of illegal pod-mod disposable e-cigarettes. Tob Control.

[22] Hendlin YH, Bialous SA (2020). The environmental externalities of Tobacco manufacturing: a review of Tobacco industry reporting. Ambio.

[23] Scheiby K. Are disposable vapes bad for the environment? Greenpeace; 2023 [updated 24 July 2023. Available from: https://www.greenpeace.org.uk/news/are-disposable-vapes-bad-for-the-environment/.

[24] Mahase E (2023). Paediatricians call for ban on disposable e-cigarettes as child vaping rises. BMJ.

[25] World Health Organisation (2021). Compendium of WHO and other UN guidance on health and environment.

[26] Munoz V, Sy DK, Syam N, Velasquez G, Yu V (2013). Financial resources for implementation of Tobacco control measures: potential of innovative financing.

[27] Callard C (2010). Follow the money: how the billions of dollars that flow from smokers in poor nations to companies in rich nations greatly exceed funding for global Tobacco control and what might be done about it. Tob Control.

[28] European Union. Directive (EU) 2019/904. European Union; 2019.

[29] Government explores next. steps to clean up tobacco litter in England [press release]. UK Goverment2021.

[30] Hendlin YH (2018). Alert: Public Health Implications of Electronic Cigarette Waste. Am J Public Health.

[31] UK Government (2019). Advancing our health: prevention in the 2020s.

[32] HM Government. Advancing our health: prevention in the 2020s – consultation document 2019 [Available from: https://www.gov.uk/government/consultations/advancing-our-health-prevention-in-the-2020s/advancing-our-health-prevention-in-the-2020s-consultation-document.

[33] The Scottish Government (2013). Creating a Tobacco-Free Generation: a Tobacco Control Strategy for Scotland.

[34] Cancer Intelligence Team. Smoking prevalence projections for England, Scotland, Wales and Northern Ireland, based on data to 2018/19. Cancer Research UK; 2020. [.

[35] Braun V, Clarke V, Thematic. analysis. APA handbook of research methods in psychology, Vol 2: Research designs: Quantitative, qualitative, neuropsychological, and biological. APA handbooks in psychology®.DOI: 10.1037/13620-004. Washington, D.C., USA: American Psychological Association; 2012. p. 57–71.

[36] Public Health England. Fiscal and pricing policies: evidence report and framework. Public Health England; 2018.

[37] Dobbie F, Purves R, McKell J, Dougall N, Campbell R, White J (2019). Implementation of a peer-led school based Smoking prevention programme: a mixed methods process evaluation. BMC Public Health.

[38] Marshman B, Wolf K, McCausland K, Daube M, Jancey J. Tobacco companies, corporate social responsibility and the use of third-party awards: a framing analysis. Tobacco Control. 2023;10.1136/tc-2022-057854:tc-2022-057854. 10.1136/tc-2022-057854 37369562

[39] Hirschhorn N (2004). Corporate social responsibility and the Tobacco industry: hope or hype?. Tob Control.

[40] Herzog C, Ali C (2015). Elite interviewing in media and communications policy research. Int J Media Cult Politics.

[41] Cairney P (2012). Complexity theory in political science and public policy. Political Stud Rev.

